# Sodium

**DOI:** 10.1016/j.advnut.2025.100409

**Published:** 2025-03-12

**Authors:** Pasquale Strazzullo, Veronica Abate

**Affiliations:** 1 Federico II University, Naples, Italy; 2 Department of Clinical Medicine and Surgery, Federico II University, Naples, Italy

**Keywords:** sodium, salt, food sources, dietary reference values, healthy habits, cardiovascular prevention

## Abstract

Sodium is the major cation of extracellular fluid (ECF) and, because of its osmotic action, is involved in the regulation of ECF volume and blood pressure. The ingested sodium is almost completely absorbed by the intestine. Circulating sodium is filtered by the glomeruli and its renal tubular handling is responsible for the maintenance of sodium and water balance. Sodium deficiency is rare and occurs only in some medical conditions. High dietary sodium intake is associated with ECF volume expansion and is a leading risk factor for hypertension and cardiovascular diseases; it also adds to risk of gastric cancer, nephrolithiasis, reduced bone mineral density, and osteoporosis. Salt added while cooking and eating, the amount added during food transformation, and that occurring naturally in foods contribute to the dietary sodium intake. Additional small amounts of sodium may be occasionally acquired through oral or parenteral medications. The National Academy of Science, Engineering and Medicine set an adequate intake of 1.5 g and a chronic disease risk reduction intake of 2.3 g of sodium per day for the adult population. The European Food Safety Authority and the World Health Organization set a standard dietary target for sodium of 2 g/d (5 g of salt). Recent studies highlighted the relevance of salt intake reduction for all-cause mortality risk and, in particular, for stroke. Sodium also appears to affect the activity of the immune system by influencing the gut microbiota composition and the macrophage and lymphocyte differentiation.

Sodium is the major cation of ECF, 1 mmol or mEq corresponding to 23 mg sodium. The mean body content in adults is 1.3–1.4 g/kg body weight. Half of the total amount of sodium is located in the ECF at a concentration of 135–145 mmol/L, 12% is found in intracellular fluids at the concentration of only ∼10 mmol/L, and 35%–40% is in the bone matrix; small variable amounts are also found in the skin and in the skeletal muscle. The concentration gradient between the extracellular and intracellular fluids is maintained by the sodium–potassium adenosine triphosphatase (Na–K pump) which transports sodium outside and potassium inside the cell, using the energy supplied by ATP. In polarized cells, such as those of the renal tubular epithelium and of the intestinal wall, sodium enters the apical surface through specific sodium channels or other transport mechanisms (for example, cotransport with other substrates, as phosphates, amino acids, glucose, and galactose), and is extruded through the basal surface into the adjacent capillaries by the Na–K pump.

Sodium and water balance in the body are strongly connected and are finely maintained by the kidneys. About 90% of the ingested sodium is absorbed in the distal segments of the small intestine and in the colon. In the kidneys, the sodium filtered by the glomeruli is almost completely reabsorbed in the proximal convoluted tubule, thanks to the action of angiotensin II and norepinephrine, and in the distal convoluted tubule, thanks to aldosterone and insulin. By contrast, dopamine, cyclic adenosine monophosphate, the cardiac natriuretic peptides, and prostaglandins exert a natriuretic effect. A small variable amount of sodium is lost through feces and sweat, depending on dietary sodium intake.

Sodium is principally involved in the regulation of BP and of electrolyte/water balance. Its role is crucial for maintaining ECF volume because of its major osmotic action. The concentration gradient between ECF and intracellular fluid contributes to the excitability of muscle and nerve cells and to the transport of nutrients through plasma membranes [1].

## Deficiencies

A clinically relevant sodium deficit in healthy individuals is extremely rare even with diets poor in sodium. Hyponatremia, that is, a plasma sodium concentration below 135 mmol/L, is indicative of an alteration of sodium and water homeostasis, but not necessarily of a reduced body sodium. A true sodium deficiency can occur however in several medical conditions, such as excessive sweating, extensive burns, chronic diarrhea, uncontrollable vomiting, excessive intake of diuretics or continuous gastric suction, and in case of sodium-losing kidney disease or of severe adrenal insufficiency [1].

## Diet Recommendations

The available data suggest that the sodium minimum intakes that prevent deficiency signs or symptoms are very low. However, because of the difficulties in the performance and interpretation of balance studies, most national and international agencies have found it unpractical to define a sodium average requirement and have rather set an age-specific adequate intake (AI) for the healthy population, corresponding to a moderate intake of sodium, compatible with a varied diet and a healthy lifestyle. The same authorities have also generally established an age-specific standard dietary target (SDT), that is, a suggested maximum level of intake for the prevention of cardiovascular disorders and other chronic degenerative diseases. The AI values indicated by most national agencies range from 0.5 to 2 g /d (1.25–5 g of salt). In 2019, National Academy of Science, Engineering and Medicine set an AI of 1.5 g/d and a chronic disease risk reduction intake of 2.3 g/d of sodium (3.75 and 5.8 g of salt, respectively) for adult individuals: these intake levels are extrapolated to children and adolescents based on the respective energy requirements [2]. In the same year, European Food Safety Authority set a single level of AI/SDT of 2 g/d of sodium (5 g of salt), the same level recommended by the WHO [3]. In 2013, the WHO Member States committed to implement national policies aiming at reduction of population sodium intake by 30% by the year 2025: because in most countries a large proportion of sodium intake comes from processed food, the WHO has recently developed benchmarks for sodium content in most food categories in an effort to support the action of national health institutions in favor of industrial food reformulation [4].

## Food Sources

Dietary sodium intake (1 g of sodium corresponding to 2.54 g of salt) is the sum of the generally small amounts of sodium/salt present in natural foods, the amounts added while cooking and eating, and the often larger amounts added to many foods upon their industrial processing. Additional small amounts of sodium may be occasionally acquired through oral or parenteral medications.

A systematic review including 80 studies from 34 countries reported a daily sodium intake in the adult population ranging from 2.1 to 6.2 g/d. The sources of sodium intake are commonly identified as “discretionary” (from the salt added to food in the kitchen or at the table) and “non-discretionary” (the sodium naturally present in foods and the one added upon the industrial food transformation), the latter being mainly in the form of sodium chloride with about one-tenth being in the form of sodium glutamate, bicarbonate, etc.

Foods naturally very low in sodium (<20 mg/100 g, with few exceptions) are fruits, vegetables, oils, and cereals. Unprocessed meat and fishery products contain from 40 to 120 mg/100 g (but some shellfish contain ≤500 mg/100 g). Whole milk contains ∼50 mg/100 g. The sodium content of processed foods varies depending on the amount of salt added during their preparation: for example, bread may contain from only traces to several hundred milligrams of sodium per 100 g. The sodium content of some processed traditional meats and cheeses is extremely high (≤2500 mg/100 g) and so also is that of several frozen foods (≤700 mg/100 g).

A few food groups, including bread and bakery products (25%–40%), cereals and grains (≤24%), meat and dairy products, are the main contributors to daily sodium intake in most countries worldwide. Meat products provide particularly high amounts of sodium (≤31%) in the United States. Dairy products account for ≤15% of daily sodium intake in Argentina and New Zealand, whereas in Japan an impressive amount of sodium (44%) comes from sauces and dressings.

The proportion of discretionary sodium intake is also extremely variable across countries depending on local culinary habits, with a tendency to be higher in countries with a lower level of economic development [5]. Total daily sodium intake also seems significantly higher in social groups with lower levels of occupational and educational attainment.

## Toxicity

Several cases of acute toxicity from massive ingestion of salt (≤10 g in children and 25 g in adults) were associated with a mortality risk of 50% because of neurological damage from acute hypernatremia. In pathological conditions, such as heart failure, decompensated liver cirrhosis, and renal failure, a high dietary sodium intake may induce a dangerous ECF volume expansion. Even among clinically healthy subjects, a high habitual sodium intake is a leading risk factor for hypertension and cardiovascular disease in most countries [6]. Furthermore, a high dietary salt intake was associated with a higher risk of gastric cancer, nephrolithiasis, reduced bone mineral density, and osteoporosis [7].

## Recent Research

Recent experimental and clinical studies have highlighted important effects of sodium intake on the immune system, by influencing the differentiation of macrophages and lymphocytes and by affecting the composition of gut microbiota [8].

Several meta-analyses of randomized controlled trials (RCT) have confirmed the beneficial effect of sodium reduction on BP in both hypertensive and normotensive individuals [6]. In particular, a meta-analysis of RCT has further indicated that the relationship between sodium intake and BP is linear starting from an intake as low as 0.4 g/d [9]. The Trial of Hypertension Prevention has detected a positive association between habitual sodium intake and all-cause mortality in a large sample of American adult population followed for a median 24 y. Previous meta-analyses of prospective studies had shown that higher salt intake is associated with a greater risk of stroke. Some studies suggesting a J-shaped instead of a linear association between sodium consumption and health outcomes have been criticized by the majority of experts because of major methodological weaknesses such as inaccurate assessment of sodium intake and strong risk of statistical confounding and reverse causality [10].

## Author contributions

Both authors read and approved the final manuscript.

## Funding

The authors reported no funding received for this study.

## Conflict of interest

The authors report no conflicts of interest.
